# Arterial Embolization and Methylene Blue Injection into the Aberrant Artery in Two Infants with Intralobar Sequestration

**DOI:** 10.1055/s-0042-1757570

**Published:** 2022-10-10

**Authors:** Anna Ayako Accarain, Marc Laureys, Luc Joyeux, Nasroola Damry, Henri Steyaert, Helena Reusens

**Affiliations:** 1 Department of Surgery, Université Libre de Bruxelles, Brussels, Belgium; 2 Department of Radiology, Brugmann University Hospital, Brussels, Belgium; 3 Department of Pediatric Surgery, Hopital Universitaire des Enfants Reine Fabiola, Bruxelles, Belgium

**Keywords:** VATS, hybrid management, intralobar sequestration

## Abstract

Bronchopulmonary sequestration is a rare congenital lung dysplasia. An intralobar sequestration (ILS) is a nonfunctional mass within the lung parenchyma without bronchial communication and with aberrant systemic arterial blood supply. Surgical resection or close observation can be proposed in the management of asymptomatic and low-risk ILS, but there is a lack of consensus. Endovascular embolization before thoracoscopic resection of ILS has been described to limit perioperative bleeding. Another technique previously reported is the injection of methylene blue in the feeding artery to macroscopically mark the sequestration from the healthy lung. In that way, a nonanatomical resection can be performed instead of a lobectomy without the risk of leaving abnormal lung tissue in place. We describe the first two cases of these two techniques combined: a 3-year-old girl with an ILS in the right lower lobe with an artery originating from the abdominal aorta, and a 14-month-old girl with an ILS in the right lower lobe with an artery coming from the celiac trunk.

The combination of embolization and injection of methylene blue in the aberrant artery leads to a clear macroscopic demarcation of the blue-colored ILS from the healthy lung parenchyma and allowed safe nonanatomical resection of the ILS without risk of bleeding or compromising normal lung tissue.

## Introduction

Bronchopulmonary sequestration (BPS) is a rare congenital lung dysplasia which can exist as extra- or intralobar sequestration (ILS). ILS consists of nonfunctional lung parenchyma without bronchial communication and with an aberrant systemic arterial blood supply. Unlike the extralobar sequestration, an ILS shares the same pleura with the rest of the lung. 1

Since ILS is known to cause recurrent pulmonary infection and hemoptysis, early and prophylactic surgery is offered especially for symptomatic or high-risk ILS. A high-risk ILS consists of a large sequestration with possible mass effect, or if another diagnosis is suspected (pleuropulmonary blastoma). 2 Hybrid lesions, synchronous lesions of congenital pulmonary airway malformations (CPAM) and BPS, can consist of anatomical BPS with histological features of CPAM or CPAM-affected lobes with an accessory systemic vascular supply. CPAM carries a risk of infection and eventual malignant transformation. 3 Surgery is mostly recommended to confirm the diagnosis on anatomopathological analysis. 4 For ILS, often a lobectomy is proposed because abnormal ILS tissue could be difficult to distinguish from healthy lung tissue. The debate between lobectomy and nonanatomical resection of ILS is still ongoing. 5

Minimal invasive surgery such as video-assisted thoracoscopic surgery (VATS) for ILS is safe, and few long-term comorbidities are associated. 6 Compared with thoracotomy, VATS induces less postoperative pain with a reduced length of hospital stay, better cosmetic results, and less musculoskeletal deformities. 7 8 9 Thoracoscopic resection of ILS is safe and efficient, and has been used for many years without embolization or blue coloration. 10 For asymptomatic and low-risk ILS, a consensus on the correct management does not exist. 11

New techniques have emerged in the treatment of ILS, such as endovascular embolization of the feeding artery before the actual removal of the mass, which decreases the perioperative risk of bleeding. 11 12 13 14 Also previously reported is the injection of methylene blue in the aberrant artery to make a clear and macroscopic distinction between healthy tissue and ILS. 15 16 This coloration allows for a complete resection without the need of a lobectomy, therefore sparing healthy lung parenchyma. Furthermore, nonanatomical resection of ILS has a comparable complication rate and does not carry a higher risk of residual disease compared with lobectomy. 17 18

In this article, we describe the combination of embolization and methylene blue injection of the aberrant feeding artery followed by VATS resection of ILS in two patients.

## Case Report

**Fig. 1:**
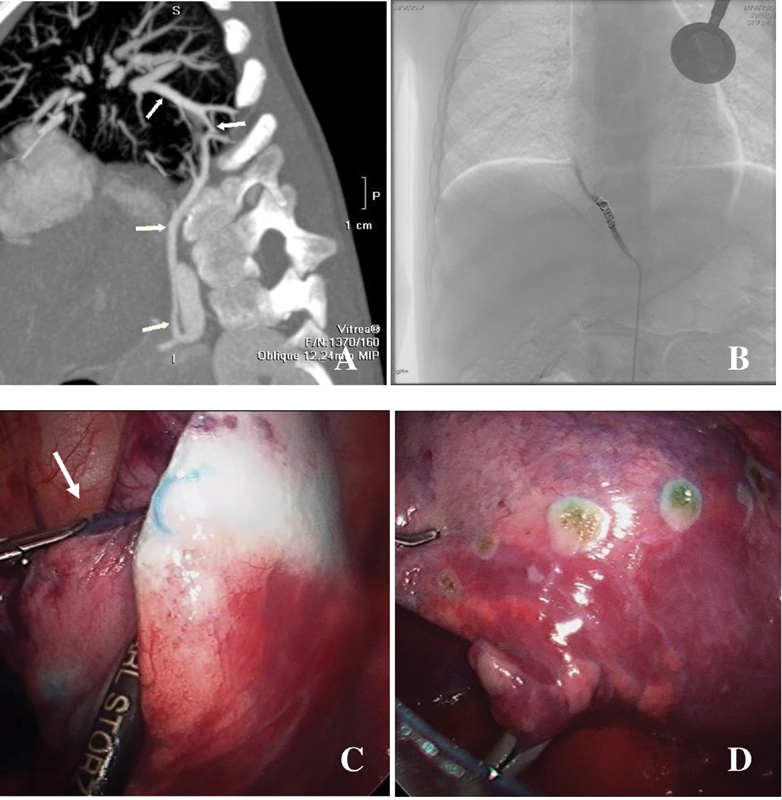
3-year-old patient. ( **A** ) Preoperative chest CT with contrast; white arrows (below): aberrant feeding artery coming from abdominal aorta, white arrows (up): venous return. ( **B** ) “Sub”-embolization with coil, correct timing for methylene blue injection into the aberrant artery. ( **C** ) Thoracoscopic view; white arrow: endoscopic injection needle placed in the embolized artery. ( **D** ) After electrocoagulation marking of the sequestration and before resection. CT, computed tomography.

**Fig. 2:**
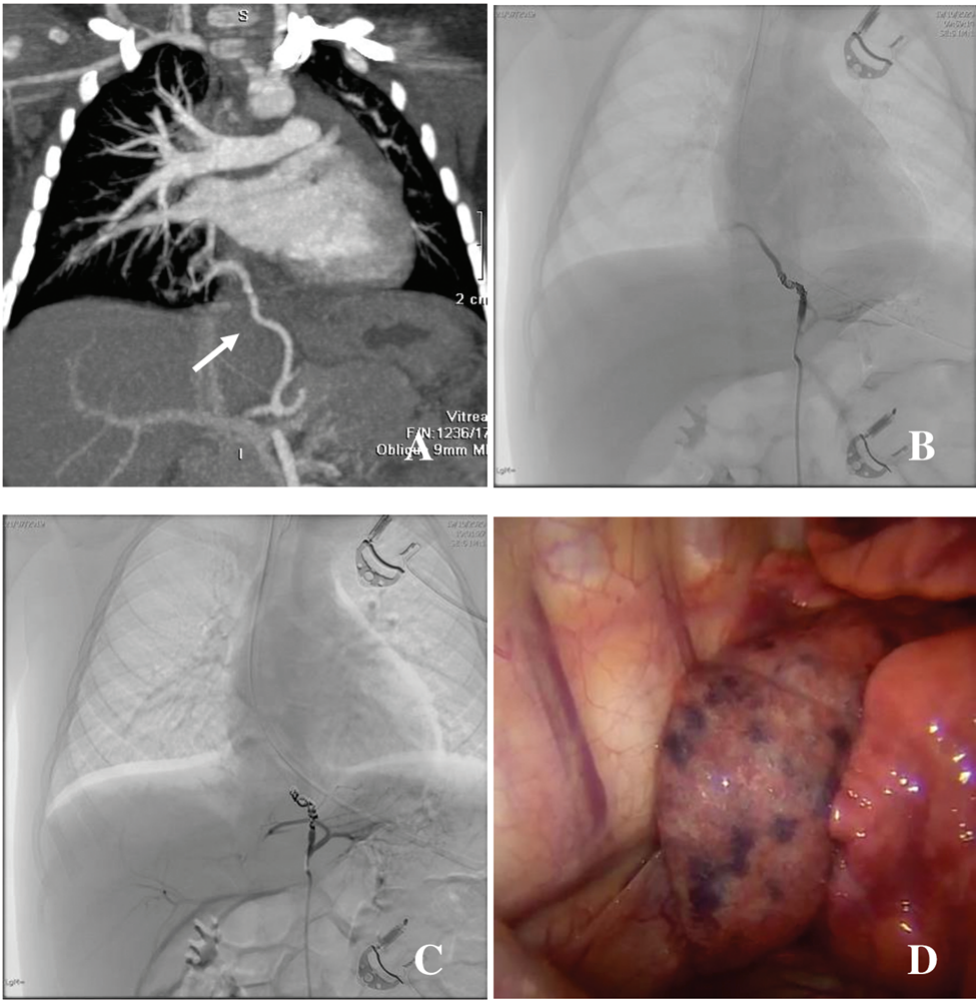
1-year-old patient. ( **A** ) Preoperative chest CT with contrast; white arrow: aberrant feeding artery coming from celiac trunk. ( **B** ) “Sub”-embolization with coil, correct timing for methylene blue injection into the aberrant artery. ( **C** ) Complete embolization with coil (no downstream blood flow). ( **D** ) Intraoperative view, blue coloration of the sequestration and clear demarcation of healthy tissue, before marking and resection. CT, computed tomography.

A 3-year-old (12.5 kg) and a 14-month-old (10 kg) female patients were referred to our hospital for management of ILS. Both cases had been diagnosed prenatally, and both patients had been asymptomatic since birth. A contrast-enhanced computed tomography scan of the chest was performed to complete the work-up. The mass localization in the lower lobe and its radiologic characteristics confirmed diagnosis of an ILS in both cases. The feeding artery was identified as originating from the abdominal aorta in the 3-year-old girl and from the celiac trunk in the 1-year-old girl ( Figs. 1 and 2 ).

The procedure and risks were explained to the parents and an informed consent for this combined technique (radiologic embolization, methylene blue injection, and surgery) was signed preoperatively. Both patients underwent the same surgical procedure, which was executed in a hybrid theater room. Conventional endotracheal intubation was used in both cases. Standard intraoperative monitoring included electrocardiogram, direct measurement of arterial and central venous pressure, pulse oximetry, continuous monitoring of temperature, and arterial and central venous blood gases.

First, our interventional radiologist performed an embolization of the aberrant artery by endovascular coiling via the right femoral artery. Once the coils had been introduced, 10 mL of diluted methylene blue (1 ampoule of 10 mg/1 mL methylene blue diluted with physiologic serum) was injected within the artery ( Figs. 1 and 2 ). The timing of the methylene blue injection was important since too early injection would lead to inefficient coloration of the mass, and in the same way, too late injection after a complete stop of blood flow into the mass would also give an inefficient coloration. In our first patient we injected too late, and we had to reinject a small amount of methylene blue directly in the feeding vessel with an endoscopic needle to have sufficient coloration during the resection ( Fig. 1 ). We also performed electrocoagulation marking of the mass before the disappearance of the blue coloration due to preserved venous drainage ( Fig. 1 ).

After the endovascular step, we positioned the patient in left lateral decubitus and proceeded to resection of the mass by VATS, using the 3 mm Bolder sealer ( Figs. 1 and 2 ), 3 mm laparoscopic instruments, and a 5 mm 30° camera. Carbon dioxide was used for insufflation and pressures were kept below 5 mm Hg. In the first case, we had some air leakage after the removal of the specimen, so a 14 Fr thoracic drain was placed and she stayed one night in the intensive care unit for observation. The leakage resolved spontaneously and the chest drain was removed on day 1 postoperatively. In the second case, we did not leave a drain.

The pathological analysis of the ILS resection confirmed diagnosis in both patients. Postoperative evolution was favorable for both children. The 1-year-old girl was discharged on the first postoperative day and the 3-year-old girl on the fourth postoperative day. The older girl had a longer stay because of the thoracic drain with secondary pain, and she also had ongoing difficulties with weight gain warranting longer surveillance of her food intake after surgery. Postoperative follow-up (FU) at 10 days and 3 and 12 months revealed no complications and both patients had a normal chest radiography.

## Discussion

Prophylactic surgery for ILS management is still recommended in most centers to prevent infection or hemoptysis and to confirm the diagnosis on histopathology, especially since thoracoscopy is safe and without long-term comorbidities. 10 It is preferred to do the surgical resection between 6 months and 1 year of age, before the occurrence of pulmonary infection, which could cause local inflammation leading to more difficult surgery. 7 19 20 In our two cases, the patients were asymptomatic and referred to our department at a later age.

Once surgery is proposed, a minimal invasive approach is nowadays preferred because of reduced postoperative pain and shorter hospital stay, and prevention of long-term musculoskeletal consequences of a thoracotomy. 9 21

Methylene blue is a dye used in different settings such as oncologic and intestinal surgeries. 22 23 It is a safe drug when used in therapeutic doses (<2 mg/kg). Contraindications include patients with severe renal insufficiency or hypersensitivity to it. Glucose-6-phosphate dehydrogenase deficiency is a relative contraindication. Toxicity has been reported, especially in neonates. 16 It should be avoided in all premature infants because of its potential life-threatening toxicity. 24

Methylene blue injection in the aberrant artery provides a macroscopic clear distinction between healthy and abnormal tissues to perform a targeted resection of the mass. We recommend marking the mass by electrocoagulation after the methylene blue injection because the discoloration disappears after a few minutes because of the remaining venous drainage.

The embolization takes away the risk of intraoperative bleeding, making it possible to undertake safe and complete resection sparing normal tissue. The most important limit of this technique is the availability of an interventional radiologist and a hybrid operating theater. Ideally, this operation should be performed in a hybrid operating room to avoid moving the patient under general anesthesia from the radiology department to the operating theater.

This is, to our knowledge, the first report of two cases of ILS managed with a hybrid technique using endovascular embolization and methylene blue coloration before minimally invasive lung-sparing surgery. Both patients are in perfect health and no complications occurred at 1 year FU. This combined technique leads to a safer resection with less risk of bleeding and healthy lung tissue preservation without compromising resection margins.

## Notes

New Insights and the Importance for the Pediatric Surgeon Two cases of intralobar sequestration managed with a hybrid technique (endovascular embolization and methylene blue coloration followed by minimally invasive surgery) leading to a safer resection with less bleeding risk and healthy lung tissue preservation.
